# Oxygen uptake at the ocular surface in diabetic animals is impaired in response to central corneal injury

**DOI:** 10.1016/j.exer.2025.110384

**Published:** 2025-04-09

**Authors:** Ana M. Sandoval-Castellanos, Sun Qin, Li Ma, Fernando Ferreira, Brian Reid, Min Zhao

**Affiliations:** a Department of Ophthalmology & Vision Science, Institute for Regenerative Cures, School of Medicine, University of California, Davis, Davis, California, USA; b Department of Dermatology, Institute for Regenerative Cures, School of Medicine, University of California, Davis, Davis, California, USA; c School of Life Science, Yunnan Normal University, Yunnan, China; d Skin and Cosmetic Research Department, Shanghai Skin Disease Hospital, Shanghai, China; e Departamento de Biologia, Centro de Biologia Molecular e Ambiental (CBMA), Universidade do Minho, Braga, Portugal

**Keywords:** (1–7): cornea, Oxygen uptake, Diabetes, Wound healing, Oxygen sensor, Optical fiber sensor, Spatiotemporal profile

## Abstract

Poor wound healing is one of the most devastating complications in late-stage diabetic patients. The avascular cornea does not rely on circulation for its oxygen consumption, uptaking it mainly from the atmosphere. Previous studies demonstrated that oxygen uptake (O_2_U) in diabetic experimental animals and patients is significantly lower than in the non-diabetic condition. Our recent experiments show that upon wounding of the central cornea the O_2_U decreased across the ocular surface, followed by two increases at 6–24 h, and at 72 h, which appeared to be important for proper wound healing. It is however not known whether the two distinct O_2_U increases are maintained in diabetic ocular surface in response to corneal injury. In this study, we used an optic-fiber oxygen micro-sensor to measure O_2_U across the ocular surface of streptozotocin (STZ)- induced diabetic mice and age-matched control mice following injury to the central cornea. We found that the injury causes an immediate and substantial reduction of O_2_U across the ocular surface. O_2_U in non-diabetic corneas increases at 2–6 h post wounding (hpw), decreasing again before the second rise to peak at 72 hpw, especially at the limbus. O_2_U in the diabetic cornea decreases more markedly than that of non-diabetic control. This defective diabetic O_2_U persisted, precluding the two dynamic rises in O_2_U, leading to a failure in recovery. Altogether, our results suggest a previously unknown mechanism of a defective O_2_U response to injury in the diabetic ocular surface, which warrants further research and may lead to new therapeutic paths.

Diabetes mellitus is the leading cause of blindness in the world, significantly impacting patients’ ability to have productive lives (Ljubimov, 2017; Xu and Yu, 2011; Zhao et al., 2019). Diabetes is a metabolic disease characterized by hyperglycemia (persistent high blood sugar) which causes the overexpression of chemokines, cytokines, and other pro-inflammatory molecules as well as pro-apoptotic genes, contributing to diabetic keratopathy (DK) (Zhao et al., 2019; Buonfiglio et al., 2024; Priyadarsini et al., 2020; Liang et al., 2022)–(Zhao et al., 2019; Buonfiglio et al., 2024; Priyadarsini et al., 2020; Liang et al., 2022). DK symptoms include corneal epithelial defects, recurrent erosions, edema, ulcers, superficial punctate keratitis, neuropathy, stem cell dysfunction, microvascular alterations in the conjunctiva, and impaired wound healing (Ljubimov, 2017; Xu and Yu, 2011; Zhao et al., 2019; Buonfiglio et al., 2024). These abnormalities are caused by damage to the corneal nerves, decreased tear secretion, changes in the structural and functional composition of the cornea, accumulation of glycation products, and oxidative stress (Priyadarsini et al., 2020). The wound healing of the cornea is challenging in diabetic patients due to delayed re-epithelialization, often followed by persistent epithelial disorders, such as epithelial fragility (Buonfiglio et al., 2024; Priyadarsini et al., 2020; Chikama et al., 2007; Bu et al., 2019).

Oxygen is essential to maintain metabolic activities for the normal function of the cornea (Chhabra et al., 2009). The sources of oxygen to the cornea are mainly the environmental air and the capillaries in the conjunctiva (Chhabra et al., 2009). Oxygen is used by the cells to produce adenosine triphosphate (ATP) as an energy source (Nishida et al., 2021). Diminished oxygen supply to the cornea leads to hypoxia, hindering the normal homeostatic processes at the ocular surface (Chhabra et al., 2009). It has been established that oxygen consumption in the whole diabetic animal and human cornea is low, causing an abnormal corneal metabolism (Graham et al., 1981). In hypoxic conditions, ATP levels decrease, and glucose is metabolized in increased amounts through glycolysis, producing high levels of lactate, which leads to cornea edema. Additionally, chronic high glucose results in the decreased or dysfunctional production of ATPase (Herse and Petchell, 1998). Herse and Petchell reported low oxygen uptake (O_2_U) and ATPase activity in diabetic cornea in rabbits (Herse and Petchell, 1998). Since O_2_U measurements at the cornea is a proxy of its metabolic activity, we recently applied a noninvasive optical fiber microsensor or optrode to generate a comprehensive spatiotemporal map of O_2_U of the intact cornea in non-diabetic rats, mice, and rhesus monkeys (Sun et al., 2023). We discovered that the intact ocular surface of diabetic mice showed decreased oxygen flux at different regions of the cornea, when compared to healthy corneas (Qin et al., 2024). We demonstrated that injury to the central cornea induced unique dynamics in spatiotemporal O_2_U: 1) significant decrease in O_2_U across the ocular surface, including the limbus and conjunctiva, which are millimeters away from the injury; 2) two oscillatory decrease-increase cycles of O_2_U within 72 h of injury in mice (Ma et al., 2024).

Although it is known that diabetic patients and animals have lower O_2_U at the ocular surface, it is not known how the O_2_U at ocular surface responds to central corneal injury. Using the self-referencing scanning micro-optrode technique (SMOT) (Ferreira et al., 2020), we mapped O_2_U at ocular surface in diabetic mice and compared that to age-matched controls. The results revealed a significant defective spatiotemporal response of the ocular surface to central corneal injury in diabetic animals. Diabetic animals have a different response with loss of the oscillatory response of O_2_U to the injury. Significantly low O_2_U across the ocular surface persisted long after the injury despite being exposed to ample O_2_ supply from environmental atmosphere.

The scanning micro-optrode technique (SMOT) and automated scanning electrode technique (ASET) interface software (version LV4), purchased from Applicable Electronics, have been recently described in detail (Ferreira et al., 2020) and will be summarized here only briefly. The SMOT is composed of a fiber-optic microsensor, amplifier, and 3D micro-positioner with computer control. The fiber-optic probe is tipped with an O_2_-sensitive fluorophore. A fluorescent light source excites the fluorophore (through the fiber-optic cable) with blue green light (λ = 505 nm). Upon collision with O_2_, the excited fluorophore is quenched, therefore affecting emission. The rate of fluorescence quenching is therefore proportional to the O_2_ concentration (pO_2_). Calibration of the SMOT system in saline with 0 % O_2_ and air saturated (20.95 %) O_2_ is performed before and after cornea measurements. The pO_2_ and O_2_ flux are calculated in real time by the ASET software during cornea measurements and calibrations.

Under a microscope the optrode was carefully moved to about 10 μm from the cornea surface. After waiting a few minutes for the optrode signal to stabilize, the recording began, with the ASET software automatically moving the optrode 30 μm between the ‘near’ and ‘far’ positions at a frequency of 0.1 Hz, while simultaneously recording the pO_2_ at the two positions. Then, O_2_ flux is calculated from these pO_2_ values using Fick’s first law of diffusion using established equations (Ferreira et al., 2020). Before and after measurements at the cornea surface, reference readings were taken more than 1 cm away from the specimen for about 2 min to confirm average zero O_2_ flux away from the cornea. O_2_ flux was measured at the following positions relative to the wound: wound center (which matched the cornea center), wound edge (~500 μm from cornea center), periphery (~700 μm from cornea center), limbus (~1.3 mm from cornea center), conjunctiva (1.6 mm from cornea center) (Fig. 1A). At early timepoints we measured at wound edge. At later timepoints, 48 and 72 hpw specifically, the wound edge was not observable as the wound healed, therefore we measured at a virtual ‘wound edge position’ at ~500 μm from the cornea center. At this timepoint, the measurement was a “second” peripheral region.

All animal procedures were approved by the Institutional Animal Care and Use Committee (IACUC) at the University of California, Davis. Eight-week-old C57/BL6 diabetic male mice and age-matched control mice were purchased from Jackson Laboratory. All male mice were used for consistency of data. A previous study found very similar O_2_ levels in the eyes of human males compared to females (*t*-test, p = 0.79) (Siegfried et al., 2011). Mice were kept in a standard 12 h light-12 h dark cycle. Group numbers were n = 6 STZ diabetics and n = 5 controls. The right eye only was wounded, the left eye was untreated. At the time of experiments mice were 9–10 weeks of age. Diabetes was induced by injection of streptozotocin (STZ, 5 mg/ml), which has been shown to generate stable diabetes on day five after administration of STZ (Graham et al., 2011). In our study, the time between the first STZ injections and shipping was 21 days. Upon arrival, mice were acclimated to the new environment for one week. Thus, at the time of experiments mice had been diabetic for at least three weeks. Blood glucose levels were measured from the tail vein using a glucometer (Accu-Chek Aviva Plus Glucometer, Roche Diagnostics) at three timepoints: before mice were shipped (by vendor), before experiments started, and after experiments finished. Glucose levels of STZ-induced mice were all above 320 mg/dl (before experiments: 403 ± 36 mg/dl; after experiments 484 ± 7 mg/dl). Glucose levels of control mice were between 150 and 215 mg/dl (before experiments: 203 ± 5 mg/dl; after experiments 180 ± 8 mg/dl). Comparing diabetic to control glucose levels for before and after experiments, respectively: p < 0.002 and p < 0.001.

Corneal epithelial wounds were made by first marking the wound edge with a sterile 1 mm diameter biopsy punch (Integra LifeSciences Corporation, York, PA), and then scraping off the epithelial layer with a sterile ophthalmologic scalpel (Beaver-Visitec, USA). For O_2_ flux measurements, mice were anesthetized with a mixture of ketamine hydrochloride (Zetamine, VetOne; 10 mg/ml) and dexmedetomidine (Dexdomitor, Zoetis; 0.1 mg/ml). Under these anesthesia conditions the eyes stayed naturally open, so no ophthalmic speculum was required. Only one eye per animal was tested as per the IACUC protocol. Mice were placed under a microscope and a well of hydrophobic silicone grease (Dow Corning) was made around the test eye and filled with artificial tear solution as measuring medium (BSS + saline, Alcon Laboratory). The untested eye received Soothe sterile lubricant eye ointment (Bausch & Lamb) to keep the cornea moist. The test eye was allowed to equilibrate for 5 min before measurements began. At the end of the study all mice were humanely euthanized by carbon dioxide inhalation and cervical dislocation as per the IACUC protocol.

Data are shown as mean ± standard error of the mean (S.E.M.). Differences between samples were compared using Student’s t-test, and statistical significance accepted at 95 % confidence limits (p < 0.05). All data analysis and statistics were done using Excel (Microsoft). Graphs were made using GraphPad Prism (version 10.3.1). MATLAB (Mathworks, Inc. USA) was used to generate schematic heatmaps of oxygen uptake at the cornea.

O_2_U at the ocular surface of both diabetic mice and control mice dropped significantly following wounding to the central cornea. The O_2_U in diabetic animals dropped even further (Figs. 1 and 2). For consistency and cross-comparisons with our previous studies, we focus on the corneal wound center, wound edge, periphery, limbus and the sclera conjunctiva (Fig. 1A) (Sun et al., 2023; Qin et al., 2024; Ma et al., 2024; Ferreira et al., 2020). As a control, we used wounded corneas in non-diabetic (normal) age-matched mice. We measured the O_2_U in intact healthy and STZ-induced diabetic corneas. Then, after a 1 mm wound was made at the central cornea, we measured the oxygen flux at 0, 1, 2, 6, 24, 48 and 72 hpw at the aforementioned positions. In the following paragraphs, first, we statistically compare intact versus wounded oxygen fluxes, then, we compare diabetic versus healthy oxygen fluxes.

O_2_U has two oscillatory increases and returns to the intact baseline value in wounded normal corneas in 72 h. At the wound center of normal corneas, oxygen flux decreased significantly at 2 hpw (p < 0.006), and then increased, reaching intact levels at 72 hpw (p > 0.05) (Fig. 1B). Similarly, at the wound edge, oxygen flux decreased significantly at 6 and 24 hpw (p < 0.05 and p < 0.003, respectively), returning to baseline values at 72 hpw (p > 0.05) (Fig. 1C). O_2_U slightly decreased at the periphery, limbus and conjunctiva after injury, returning to pre-wounding oxygen flux values at 72 hpw (p > 0.05) (Fig. 1D–F). The overall trend of O_2_U, i.e., the pooling of all its consumption in healthy, normal, wounded corneas highlights the lowering of O_2_U compared to intact pre-wounding values. Then, O_2_U recovered to baseline values at 72 hpw. Together, this data suggests that when wound healing is nearing its completion, the oxygen consumption is restored in normal corneas following two distinct oscillatory increases of O_2_U most obvious at the limbus (Figs. 1E and. 2B upper panel).

Wounded diabetic ocular surface has significantly decreased O_2_U at the periphery, limbus and conjunctiva. O_2_U at the wound center and wound edge decreased significantly at 6 and 24 hpw (p < 0.003 and p < 0.004 for center; p < 0.004 and p < 0.008 for edge, respectively) in comparison to pre-wounding levels. Then, O_2_U increased in these positions, approaching the intact baseline values at 72 hpw (p > 0.05 for both), despite a substantial average difference (Fig. 1B–C). O_2_U at the periphery decreased significantly compared to the baseline values (p < 0.04 at 6 hpw and p <0.01 at 24 hpw), failing to return to these values at 72 hpw (p < 0.02) (Fig. 1D). Oxygen flux at the limbus robustly decreased at 6 hpw, having statistical difference at 24, 48 and 72 hpw (p < 0.03, p < 0.04, and p < 0.03, respectively), failing to return to intact baseline values (Fig. 1E). At the conjunctiva, oxygen flux decreased significantly at 6, 24, 48 and 72 hpw (p < 0.03, p < 0.01, p < 0.02, and p < 0.05, respectively) compared to pre-wounded values (Fig. 1F). The overall O_2_U in wounded diabetic corneas reveals the decreased oxygen consumption for 72 h and its failure to return to intact baseline levels, unlike that in non-diabetic animals (Fig. 2). The diabetic animals lost the dynamic responses, i.e. the oscillatory increases in O_2_U, most notably at the limbus (Figs. 1E and. 2B).

Diabetic ocular surface has impaired O_2_U after wound healing in comparison to normal corneas. While oxygen flux decreased significantly in diabetic corneas compared to healthy controls at 1, 6 and 24 hpw and at 1 and 48 hpw in the wound center and wound edge, respectively, at 72 hpw O_2_U was not significantly different (Fig. 1B–C). Interestingly, as we moved away from the wound, we found a significant deficiency in O_2_U at the periphery, limbus and conjunctiva of the wounded diabetic cornea compared to normal cornea. At the periphery, O_2_U at 48 and 72 hpw decreased significantly (p < 0.05 and p < 0.001, respectively) in comparison to their non-diabetic counterparts (Fig. 1D). Intriguingly, at the limbus, oxygen flux decreased significantly at 6, 24, 48 and 72 hpw (p < 0.01, p < 0.01, p < 0.01, and p < 0.05, respectively) in comparison to normal controls (Fig. 1E). Additionally, O_2_U decreased at the conjunctiva of diabetic animal following wounding the central cornea, at 24 (p < 0.01), 48 (p < 0.001) and 72 (p < 0.01) hpw (Fig. 1F).

After wounding, O_2_U in the diabetic ocular surface at different positions was significantly lower when compared to normal controls. Moreover, within 72 hpw, we observed that the oxygen flux across the diabetic ocular surface decreased consistently, failing to recover to intact baseline values, as highlighted by the schematic representation in Fig. 2A and heatmap of oxygen flux magnitudes in Fig. 2B. Altogether, these data show a significant diabetic defect of O_2_U in the ocular surface, mostly in the periphery, limbus and conjunctiva. Importantly, the unique O_2_U dynamics, i.e. oscillatory increases, one at 2 hpw and the other at 72 hpw were lost in diabetic animals, indicating impairment of O_2_U across the ocular surface despite ample O_2_ supply, which is likely due a significant metabolic defect at the tissue level that impairs wound healing in diabetic corneas.

In recent studies we detailed the spatiotemporal dynamics of oxygen flux at the intact and wounded mammalian ocular surface. We found that intact healthy ocular surface uptakes oxygen in a centripetal gradient, both *ex vivo* and *in vivo* (Sun et al., 2023), which diminishes at the intact STZ-induced diabetic corneas (Qin et al., 2024). Upon injury of healthy (normal) corneas, we showed that the O_2_U decreased in the first 6 hpw, and then it returned to the intact baseline values at 72 to 168 hpw (Ma et al., 2024). Stemming from these findings, we asked whether the O_2_U is altered in wounded diabetic corneas. Poor oxygenation in diabetic tissues is normally attributed to vasculopathy where poor blood supply is the culprit (Catrina and Zheng, 2021; Huang et al., 2023). The avascular cornea offers an excellent model to probe the mechanisms, other than poor blood supply, of lower oxygen metabolism in diabetes. In this study, we used the optrode technique (Fig. 1A) and mapped the ocular surface of wounded diabetic corneas to determine the O_2_U profile across different positions over time (Fig. 1A).

We observed that O_2_U decreased across the ocular surface after central corneal injury and saw an oscillatory dynamic response of O_2_U, particularly at the limbus, before returning to preinjury values, as in our previous study (Ma et al., 2024) (Figs. 1E and. 2). The O_2_U profile in diabetic ocular surface during wound healing differs greatly from normal control corneas in spatial pattern and in the dynamic response. Injury to the central cornea caused massive reduction of O_2_U across the ocular surface in both diabetic and control animals. Non-diabetic control showed an increase 2–6 hpw, decreasing again before the second increase of O_2_U, and returning to preinjury level at 72 hpw (Figs. 1E and. 2B, upper panel). O_2_U in the diabetic corneas, however, decreased drastically and more significantly than that of non-diabetic control (Fig. 2B, lower panel). The defective O_2_U kept deteriorating and did not show the two dynamic increases in O_2_U at the limbus as in the control animals and failed to recover. In assessing our previous published results (Shen et al., 2016), it was observed that in control mice the wounds healed 50 % at 24 hpw and were almost completely healed at 48 hpw. Moreover, cornea wounds in diabetic mice healed very slowly, with only 42.6 % at 48 hpw (Shen et al., 2016). Others reported that cornea wounds healed 97 % and 77 % in normal and diabetic mice, respectively, at 72 hpw (Yang et al., 2014). Zieske and Gipson showed that in healthy corneas, incorporation of 3H leucine and 3H glucosamine (which are synthetize during migration (Zieske and Gipson, 1986) peaked at 16 hpw. 3H glucosamine and 3H leucine were present until 24 and 48 hpw (Zieske and Gipson, 1986). O_2_U in non-diabetic ocular surface largely returned to a steady state within 24 h, except that at the wound center which returned 48 h after wounding (Figs. 1 and 2). However, O_2_U in diabetic ocular surface in our experiments never returned to a steady state or pre-injury values even up to 72 hpw at periphery, limbus and conjunctiva (Fig. 1D–F). Such a long-lasting effect is more visually evident in Fig. 2.

Cornea wound healing is a complex process that comprises migration, proliferation, growth factor and cytokine production and signaling, and extracellular matrix (ECM) remodeling (Ljubimov and Saghizadeh, 2015; Huo et al., 2009). Epithelial wound healing, broadly, has two phases: a first one where cells migrate from the wound edge to the wound center; and a second phase that includes not only cell migration, but proliferation, differentiation and stratification to reestablish the epithelial layer (Ljubimov and Saghizadeh, 2015; Huo et al., 2009). Reactive oxygen species (ROS) can cause cellular damage at high concentration; however, it has been shown that a moderate ROS concentration is key for corneal epithelial wound healing (Huo et al., 2009, 2020). We recently found that decreased O_2_U at the wound center and edge, in normal murine corneas, promotes a centrifugal gradient of O_2_U from the center of the cornea to the limbal region, enabling centripetal cell migration through ROS (Ma et al., 2024). Furthermore, we also found a correlation between cell proliferation and oxygen flux at the periphery of the wounded cornea. We showed a significant increase in cell proliferation from 24 to 72 hpw, which was directly proportional to oxygen influx (r = 0.68, p = 0.043) in the periphery of the cornea (Ma et al., 2024). In the wounded mouse cornea, this spatiotemporal oxygen dynamic, ROS production and cell migration and proliferation suggest a temporal and spatial cellular responses to achieve corneal wound healing.

After injury, proliferation increases ~9 and ~2 fold in the limbus and peripheral/central cornea respectively, returning to pre wounded levels after wound closure and at 36–48 h in the limbus (Fig. 1E). Additionally, in response to injury, limbal epithelial stem cells (LESC) give rise to transient amplifying cells (TAC), which proliferate and later differentiate in response to growth factors, cytokines, integrin receptors and ECM (Ljubimov and Saghizadeh, 2015; Lavker et al., 1998; Lehrer et al., 1998; Cotsarelis et al., 1989). Following injury, insulin-like growth factor-I (IGF-I) is upregulated, stimulating the expression of IGF-I receptor in LESC, which encourages cell differentiation into corneal cells (Trosan et al., 2012).

In contrast, cornea wound healing in diabetic mice is impaired because multiple factors that are critical for the normal process are altered in diabetes. The P2X7 ion channel, which is activated by ATP, encourages cell migration. However, when P2X7 is altered, there is a decrease in actin organization and limited focal adhesion; hence, appropriate cell migration and wound healing is not achieved (Ljubimov, 2017; Minns et al., 2016; Mankus et al., 2011). Advanced glycation end products (AGEs) are the product of non-enzymatic glycosylation of proteins as a consequence of hyperglycemia in diabetes. AGEs cause formation of ROS and alterations in adhesive proteins, delaying wound healing (Ljubimov, 2017).

As previously mentioned, in our previous work, it was observed that in non-diabetic ocular surfaces, wounds healed almost completely at 48 hpw, whereas wounds in diabetic corneas only healed 42.6 % (Shen et al., 2016). It can only be hypothesized that, due to the loss O_2_U dynamics at the limbus in the wounded diabetic cornea at 48 hpw, proliferation and differentiation is decreased or altered. Additionally, differentiation in the periphery might also be hindered. Further analysis needs to be performed to understand this.

Oxygen is critical element of metabolic activity (Graham et al., 1981; Rubinstein et al., 1990) and is critical for producing ATP, the cellular energy source. ATP is used for cell proliferation and migration, for the secretion of ECM proteins and many more molecular and cellular activities for wound healing (Yeung and Dwarakanathan, 2021). As we showed previously (Sun et al., 2023; Qin et al., 2024; Ma et al., 2024), the limbus and conjunctiva are more metabolically active than the central cornea. This might be related to the activity of LESC, which reside in the palisades of Vogt in the limbus, as they are the source of epithelial renewal, and become active during wound healing (Elhusseiny et al., 2022; Yu et al., 2022).

Corneal homeostasis depends on oxygen and nutrients, which are received through the tear film from the outside, the aqueous humor from the inside, and the limbal vasculature, or limbal vascular arcade located in the limbus (Bukowiecki et al., 2017; Romano et al., 2022; Nureen et al., 2024). In contrast to other tissues, having blood vessels in the cornea interferes with sight and causes vision impairment and loss (Bukowiecki et al., 2017). Thus, the corneal angiogenic privilege, is essential for its function and is actively maintained (Bukowiecki et al., 2017).

LESC are dysfunctional in diabetic patients, causing alterations in the renewal of the epithelial layer, resulting in compromised wound healing (Ljubimov and Saghizadeh, 2015; Yu et al., 2022). This abnormal metabolic activity of LESC and epithelial cells may be related to impaired O_2_U across the ocular surface, particularly at the limbus. Significantly, our results revealed that diabetic animals lost the typical dynamics of O_2_U recovery in the days after injury, which did not fluctuate and return to pre-wound baseline values, suggesting a “vicious cycle”: low oxygen flux therefore reduced wound healing, resulting in more injuries and less oxygen at the ocular surface. Thus, the lack of oxygen in the diabetic cornea may cause the decreased wound healing response, resulting in an ocular surface that fails to maintain its O_2_U, aggravating its capacity to regenerate and further complicating ophthalmic health.

In diabetes, the vasculature is compromised. Therefore, we can only hypothesize that in the diabetic ocular surface, the metabolism of the cells in the limbus is active due to the higher oxygen flux in comparison to other regions, but that it is decreased in comparison to the normal cornea, suggesting that both the diminished oxygen flux and alteration in the vasculature play important roles during wound healing. When the ocular surface is not covered by the palpebral conjunctiva, atmospheric oxygen level is high so provides the major source for ocular surface cells (O_2_ pressure 160 mm Hg from room air vs. 75–100 mm Hg in artery blood). We can only speculate at this point that the altered consumption of oxygen and changes in the vasculature at the limbus hinders proliferation and differentiation of LESC. Therefore, further investigation is needed to understand this dynamic and complex process during wound healing in DK and the contributions from atmospheric O_2_ and vasculature O_2_.

Expression of Oxygen metabolism-related genes following corneal injury have been reported, including those involved in hypoxia response (HIF-1α, VEGF, GLUT1, LDHA, PDK1) and antioxidant defense (SOD1, SOD2, SOD3, CAT, GPX, PRDX6) (Kumar et al., 2024). These changes adapt the injured cells to altered oxygen requirements and oxygen availability, as well as managing oxidative stress, all to support healing. Future studies including in-depth analyses of oxygen-metabolism related genes, for example genes related to HIF (hypoxia inducible factor), ROS, angiogenesis, cell cycle and apoptosis will be very interesting (Pang et al., 2021; Safvati et al., 2009), to complement O_2_U measurements and provide a better insight of the mechanisms involved. Because oxygen consumption is a ubiquitous requirement for almost all cell functions, changes in O_2_U are likely to correlate with a wide variety of processes during the healing process.

Previously, we showed that there was no significant difference in O_2_U across the limbal area in the cornea of a normal mouse (Qin et al., 2024), therefore, we only measured one region in diabetic corneas, even though we were expecting to see an increase of oxygen flux at the nasal region, as the highest concentration of LESC is found there (Nureen et al., 2024). Future experiments should include additional measuring positions within the limbus to determine a detail spatiotemporal O_2_U profile during wound healing in diabetic corneas. The quadrant difference of O_2_U has been reported in human cornea at superior, central and inferior sites (Benjamin and Ruben, 1995). The authors of the report suggested that in human eyes, the upper eyelid normally covers part of the cornea, which may be the reason for the difference. In mouse cornea, a few publications suggest quadrant difference in stem cell density. For example, there appear to be more limbal epithelium stem cells in the superior temporal quadrant and lowest in the inferior nasal quadrant (Zhao et al., 2009). It is not known whether such quadrant differences are present in diabetic cornea. Following injury, limbal epithelial stem cells activate and initiate wound healing-related processes. Therefore, oxygen uptake might be higher in the superior temporal quadrant because there are more limbal stem cells there (Zhao et al., 2009). However, the overall literature leans toward no significant differences in how stem cells from different quadrants respond to injury and contribute to healing. Future studies will need to take this into consideration for a more detailed characterization of the oxygen uptake in the ocular surface following injury.

The tip size of the optrode, according to the manufacturer, is “<50 μm”. We find the tip size in the batch we used to be 20–30 μm. The optrode was placed ~10 μm (near position) from the ocular surface and then moved to 40 μm (far position). Altogether, the dimensions of the optrode and the distance between the probe and the ocular surface defines the SMOT as a spatially high-resolution system. This indicates that the bathing solution depth does not influence local O_2_U readings close to the cornea surface at the different positions we measured. We do not think the presence of bathing solution affected the readings at specific positions. The pseudo-color images rendered from a few measurements represent centrifugal O_2_U gradient and its changes following injuries in both diabetic and non-diabetic corneas. They do not necessarily indicate a homogeneous pattern of O_2_U as the pseudo-color distribution.

In this paper it was shown that the diabetic ocular surface responds to cornea injury significantly differently from non-diabetic ocular surface in the following ways: 1) more significant decreases in O_2_U at the periphery, limbus and conjunctiva following central corneal injury which did not recover and remained significantly low at 72 hpw, the longest time point in this study; 2) O_2_U at the limbus lost the dynamic increases (one after 6 hpw and another after 48 hpw) which coincide with reported cellular responses following injury (migration and proliferation). Therefore, as a diagnostic, the oxygen probe can measure intact eyes to assess cornea O_2_ metabolism in diabetes; to predict the probability of poor healing; and for injured eyes to assess how likely the recovery may progress and whether other therapies, such as providing O_2_, may help. We hope that these results also facilitate the understanding of the mechanisms of pathology in diabetic tissues.

This spatiotemporal O_2_U profile of wounded diabetic corneas provides new information about the metabolism of the ocular surface post-wounding in a widespread disease context. This profile might be used as a novel method for diagnosis and monitoring of DK. The oscillatory increases in the non-diabetic animals may suggest possible intervention points where increasing O_2_U may help healing responses of the cornea.

This study reveals an altered spatiotemporal O_2_U dynamic in the diabetic ocular surface *in vivo* in response to injury, suggesting a mechanism of abnormal oxygen metabolism other than vasculopathy in diabetic tissues. Furthermore, the difference between normal wound O_2_U recovery and diabetic non-recovery might be related to impaired diabetic wound healing. The fiber optic-based SMOT system is ultrasensitive and noninvasive, making it a promising tool for monitoring, diagnostics and therapeutic assessment.

## Figures and Tables

**Fig. 1.**
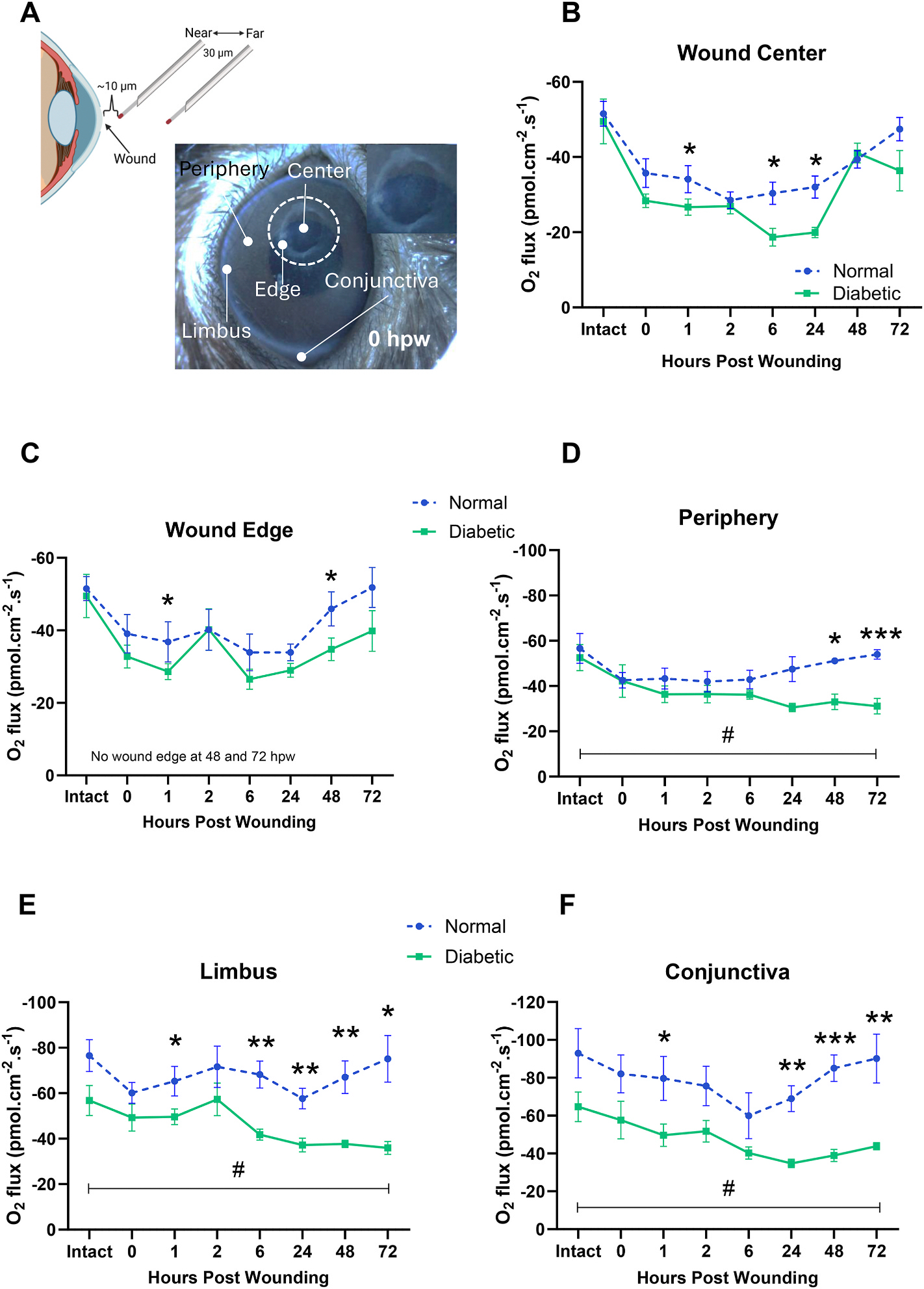
Oxygen uptake at ocular surface of diabetic mouse dropped significantly lower following cornea injury. **A.** The micro-optrode measures O_2_ uptake with high spatiotemporal resolution. The tip was positioned ~10 μm above the region of interest. The moving step between near and far position was 30 μm. Five measurement positions (wound center, wound edge, periphery, limbus and conjunctiva) are indicated. Wound (wound edge is showed as dotted white line) was healed in non-diabetic controls by the end of 2–3 days but not in diabetic corneas. Therefore, at later time points, 48 and 72 hpw specifically, the wound edge was not observable as the wound healed, therefore we measured at a virtual ‘wound edge position’ at ~500 μm from the cornea center. At this timepoint, the measurement was a “second” peripheral region. **B-F.** O_2_U before wounding and then at 0, 1, 2, 6, 24, 48 and 72 h post wounding (hpw) at wound center (B), wound edge (C), periphery (D), limbus (E) and conjunctiva (F). Oxygen uptake in both the control and diabetic animals decreased significantly from preinjury values at all measurement points after injury. The decreases in diabetic animals at all measurement points are significantly more than the normal controls respectively. The oxygen uptake in non-diabetic controls gradually recovered and returned to preinjury values, but that in diabetic animals failed to recover. O_2_U at the periphery, limbus and conjunctiva in diabetic animals never returned at the longest measurement point 72 hpw and bifurcated further from the dynamic of oxygen uptake curves more significantly from that in control animals (D, E, F). Note that the O_2_U at the limbus showed two distinct rises, which is absent in diabetic animals (E). Data expressed as mean ± S.E.M. N = 5–6. Two-tailed Student’s *t*-test. *p < 0.05, **p < 0.01, ***p < 0.001 for comparison between normal v. Diabetic at specific time point. #p < 0.05, ##p < 0.01 for comparison between intact and 72 hpw in diabetic cornea. Parts of the pre-injury data were reported in Sun et al. (2024).

**Fig. 2.**
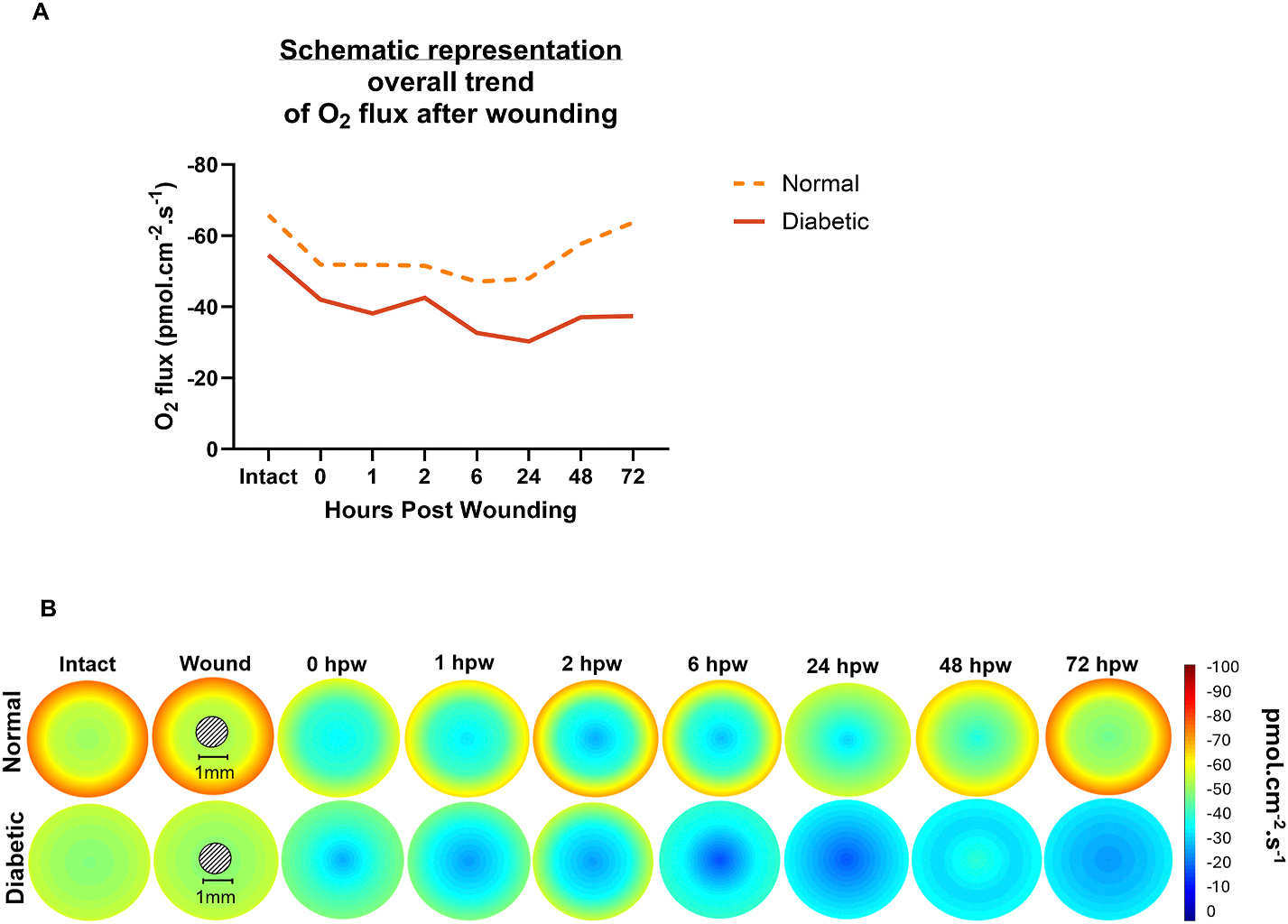
Significant defects in oxygen uptake responses following cornea injury at the ocular surface. **A.** Schematic representation of average of O_2_ uptake values measured at the center, wound edge, periphery, limbus and conjunctiva shows bifurcation of the time courses of O_2_ uptake in diabetic animals from normal non-diabetic controls. O_2_ uptake in non-diabetic controls recovered after injury, whereas diabetic animals lost the ability to recover O_2_ uptake. **B.** Pseudo color images allow visualization of the spatiotemporal profile of oxygen uptake during wound healing in normal and diabetic cornea, where red and yellow indicate high oxygen influx while blue shows lower influx. Non-diabetic control animals showed two distinct increases in O_2_ uptake (2hpw and 72hpw), whereas in diabetic corneas, oxygen uptake across the ocular surface did not show the increases (recoveries) of the conserved peaks at the limbus and conjunctiva after wound healing. In diabetic animals, the drop in O_2_ uptake was remarkably more significant, and the pattern of centripetal gradient of O_2_ uptake appeared obviously weaker. The O_2_ uptake across the ocular surface in diabetic animals strikingly plummeted at peripheral, limbus and conjunctiva, in sharp contrast to the return to normal of O_2_ uptake 72 hpw. Dark blue indicates the lowest oxygen uptake (0 pmol cm^−2^s^−1^), while dark red is the highest oxygen uptake (−100 pmol cm^−2^s^−1^).

## Data Availability

Data will be made available on request.

## Abbreviations

O_2_U: oxygen uptake
hpw: hours post wounding
STZ: streptozotocin
